# A Review of Diagnosis and Management: Persistent Cloaca Treated by a Posterior Sagittal Approach With a Normal Functional Outcome

**DOI:** 10.7759/cureus.23737

**Published:** 2022-04-01

**Authors:** Muhannad Wael, Wael M Abuarafeh, Mohammed A Lubbad, Sara Almansour, Mohammad Ghannam

**Affiliations:** 1 Medicine, An Najah National University, Jerusalem, PSE; 2 Urology, Saint Joseph Hospital, Jerusalem, PSE; 3 Urology, Shifa' Medical Complex, Gaza, PSE; 4 General Medicine, An-Najah national university, Nablus, PSE; 5 Medicine, Najah National University, Ramallah, PSE

**Keywords:** ­reconstructive surgery, pediatric reconstructive urology, congenital malformation, urology, pediatric urology, pediatric surgery, persistent cloaca, cloaca

## Abstract

Cloacal malformation (CM) is a severe, complex, and extremely rare category of anorectal and urogenital tract malformations. Prenatal diagnosis is illusory and vague; therefore, magnetic resonance imaging (MRI) is the most effective test point toward an accurate diagnosis. Thus, careful investigation and evaluation are mandatory since they could be associated with syndromes and other anomalies, including urogenital tract, vertebral, and cord abnormalities.

Despite the severity and complexity of the deformity, CM cases are curable, not desperate, and can have an excellent prognosis with great surgical correction. However, managing persistent cloaca necessitates a careful assessment because corrective surgeries require inclusive surgical planning, multidisciplinary, expert, and highly specialized medical center. In surgically repaired malformations, fecal and urinary incontinence has been a major issue, which was resolved when Dr. Pena Alberto suggested safer dissection and less harmful techniques for neurovascular structures and great functional corrected anomaly to ensure fertility and less incontinence. For improved results and prognosis on quality of life, patients should be scheduled for extended bowel training along with the clinical evaluation follow-up. In this article, we present a case successfully treated with the posterior sagittal approach, Pena operation, and anorecto-vagino-urethroplasty with feminizing clitoroplasty and highlight the value and impact of prenatal evaluation, diagnosis, and management. The rarity of the case and excellent results, including fair to normal bowel and urinary control, prompted us to report it and assert the significance of assessment, surgical management and technique, challenges, postoperative bowel training, and clinical investigation and examination.

## Introduction

Cloacal malformation (CM) is an extremely rare disease with an incidence of ~0.002% [1-3]. It is found in vertebrates, such as birds and reptiles, but not in placental mammals like humans.

Cloaca is found transiently during embryological evolution between the fourth and seventh weeks of intrauterine life when the urogenital sinus structures and gastrointestinal tracts emerge from the same origin, the endoderm. All genitourinary organs originate from the mesoderm, except for urogenital sinus structures, genital swellings, and adrenal medulla, which emerge from the endoderm.

The common chamber of the urogenital and anorectal tracts separates into hindgut and urogenital sinus. Tourneux fold and Rathke’s plicae fuse to form the urorectal septum, which divides the common chamber into the urogenital sinus and anorectal canal. Impairment and unsuccessful separation and subdivision will result in persistent cloaca malformation.

If not promptly and properly treated, the malformations are formidable, severe, and fatal. CMs are considered the most immense technical challenge to repair anatomical defects in anorectal and urogenital congenital malformations.

Surgical management is a challenging, advanced, and distinct mode of surgery, with the ultimate goal of ensuring well reconstructed functional and controlled fecal, urine, sexual organs, and future fertility. Since the posterior sagittal approach was proposed in 1980, surgical outcomes have significantly improved, and it is currently preferred over the abdominal approach. The initial treatment and definitive reconstruction of CM are the most technically challenging clinical treatment options [4].

Dr. Alberto Pena proposed the posterior sagittal technique in 1980, and in 1853, Amussat is credited with being the first individual to sew the rectum with the perineum. Hence, we present a case managed by one session posterior sagittal anorectoplasty, vagino-urethroplasty, and feminizing clitoroplasty, with fair to normal bowel and urinary control and excellent anatomical repair shape.

## Case presentation

A one-year-old girl with a known case of persistent cloaca was referred to our department of urology for definitive reconstructive posterior sagittal anorecto-vagino-urethroplasty (PSARVUP). Physical examination revealed an enlarged clitoris with a single perineal opening, while preoperative abdomino-pelvis ultrasound revealed urinary tract anomalies. The patient came with a colostomy (totally divided end colostomy and a mucus fistula to avoid passage of stool to the distal colon) done on the second day of life.

**Figure 1:**
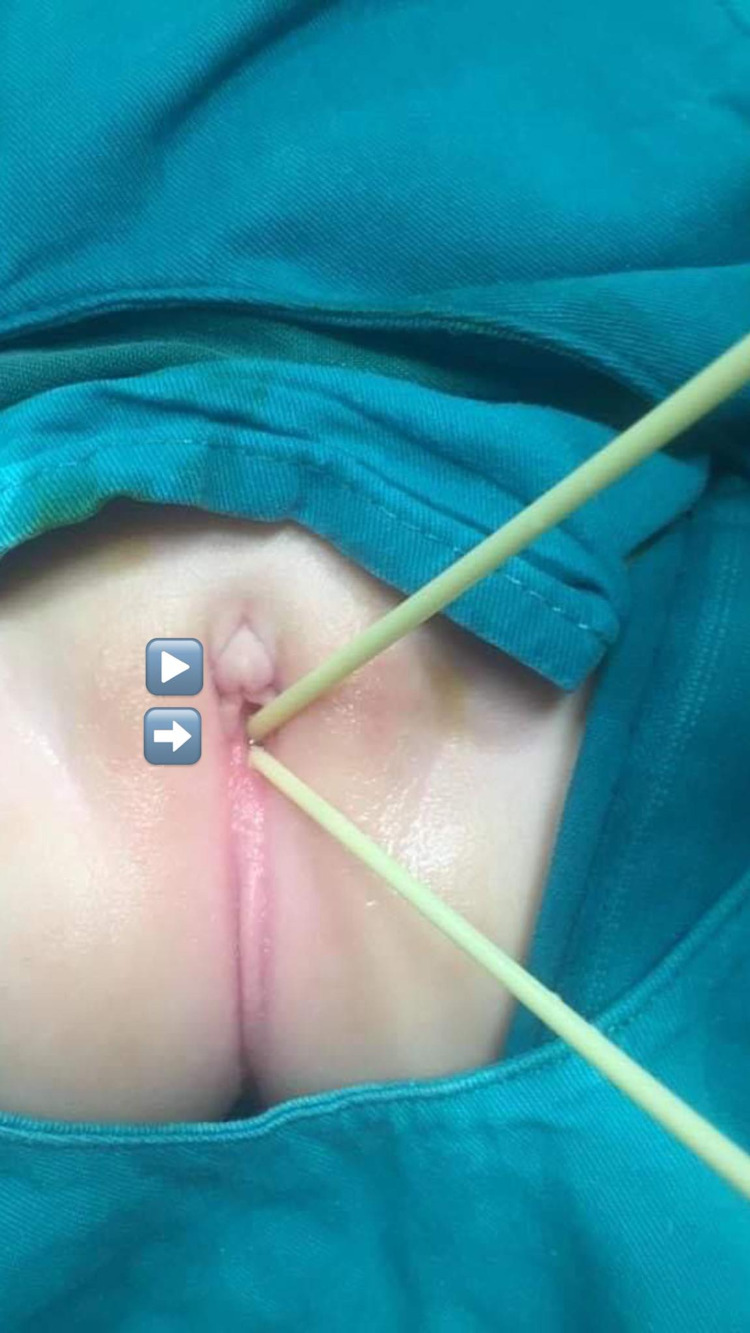
A large clitoris (arrow head) with a single perineal opening appeared, and below a double catheter (arrow) was inserted in the vagina and bladder via endoscopy.

The procedure was done at the age of one year old, with an endoscopic evaluation cystoscopy (used to evaluate the level of confluence, which was below the level of the lower border of pubic symphysis with a short common channel <3cm), genitography (provided an exact position of the confluence, and excluded the possible vaginal duplication), and the insertion of a urinary catheter, followed by vaginoscopy and the insertion of a vaginal catheter (Figure 1).

**Figure 2:**
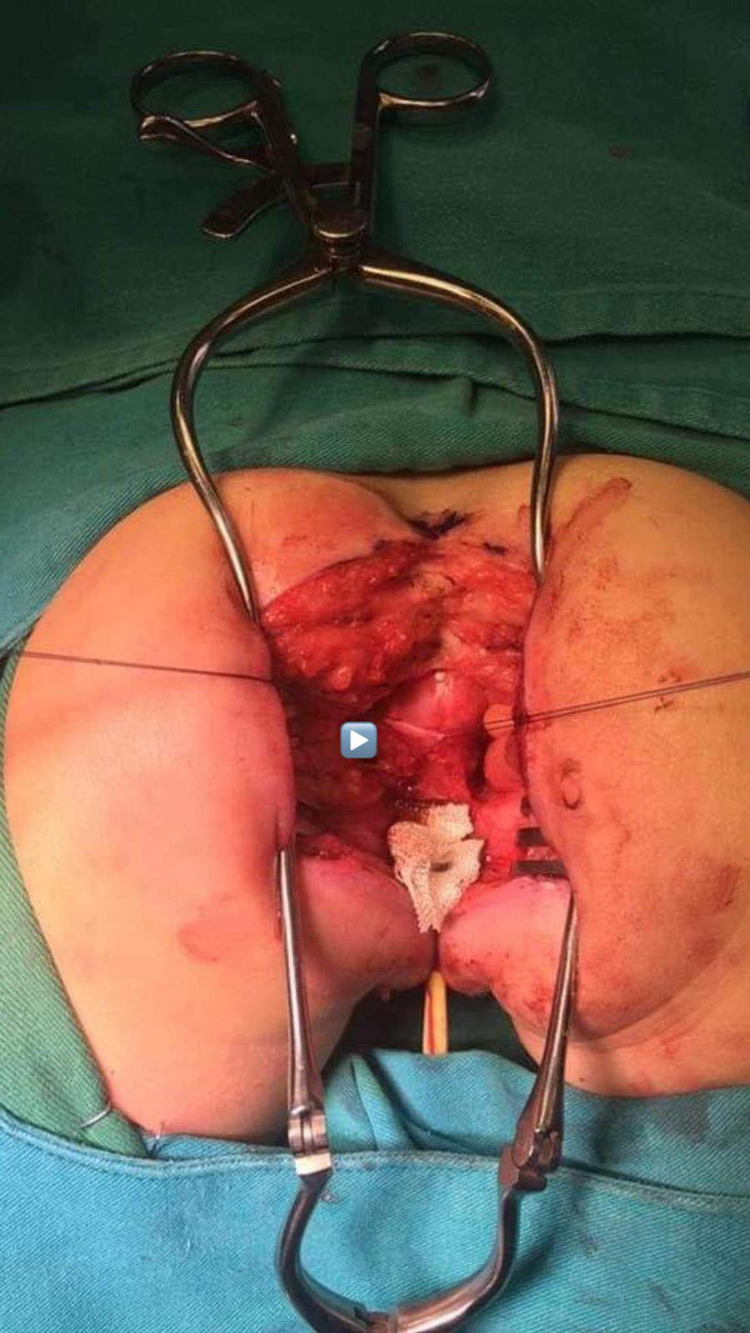
Posterior sagittal approach in a prone position with a midline incision from the coccyx and the muscular plains of the common channel (arrow) tagged with sutures.

**Figure 3:**
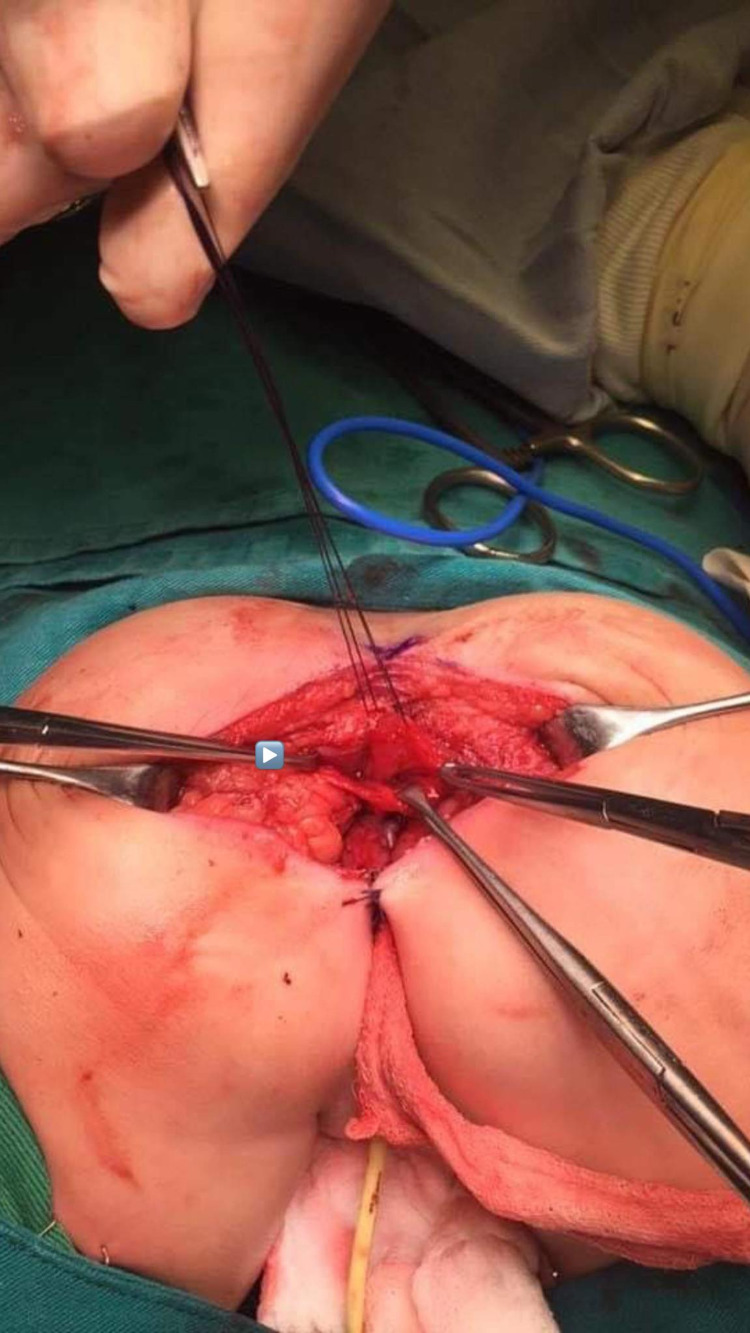
New rectum (arrow head) was done from the proximal loop of the colon, ready to be sutured with the perineum.

After putting the patient in a prone position, the posterior sagittal incision was made. The anal sphincter was identified by nerve stimulation, posterior sagittal anorectoplasty with total urinary mobilization was performed, and the Pena operation, wherein the urethra and vagina were separated as one structure (both the urethra and the vagina were mobilized together as a single unit), was conducted (Figure 2). The anorectal sphincter reconstruction, a new rectum, was performed from the proximal loop of the colon (Figure 3).

**Figure 4:**
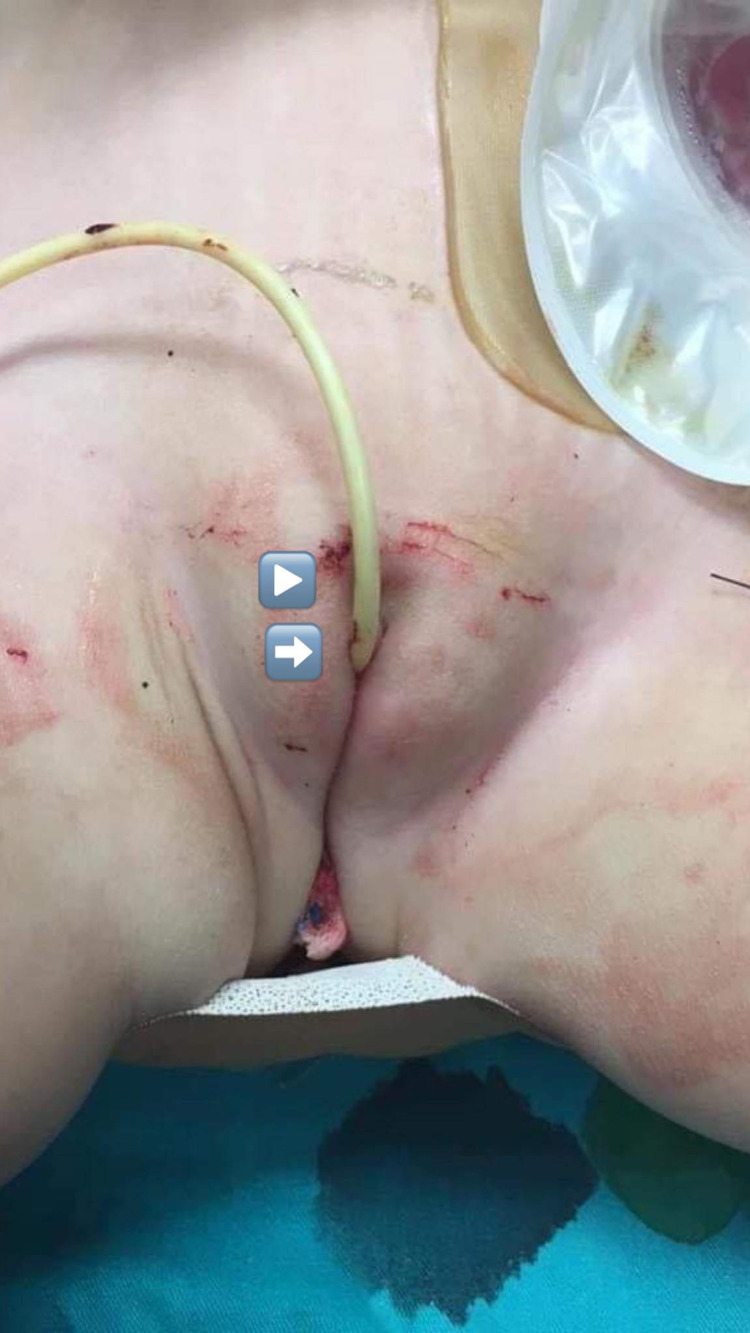
Final appearance of the clitoris (arrow head), below, the new urethra with a catheter inserted (arrow), vagina, and anus after PSARVUP with clitoroplasty. PSARVUP: posterior sagittal anorecto-vagino-urethroplasty

After shifting the patient to a supine position, vaginal and urethral walls were dissected, and vaginoplasty with urethroplasty was performed. Feminizing clitoroplasty was also performed (Figure 4).

The operative time was 160 min. Postoperative and recovery were uneventful and the patient was doing well and discharged after one week. She was scheduled for a long-term follow-up to assess her bowel and urinary control. On the second week, postoperative visit and examination revealed patent vaginal, anal, and urethral openings.

The post-operative outcomes were examined based on the following objective measures after six months and during the follow-up appointment: rectal and urinary continence, ability to evacuate the rectum and bladder spontaneously, preservation of renal integrity, and adequacy of vagina and sexual function. Our patient had no problems with his capacity to urinate or evacuate her rectum, no constipation, just infrequent episodes of diarrhea, and normal kidney function tests. However, we will need to examine our patient again at the age of four years to adequately re-evaluate and assess her urinary and rectal continence.

## Discussion

Persistent cloaca malformation is assessed as the extreme type of anorectal malformations (ARMs). “Persistent cloaca” is a severe malformation affecting females, wherein the urinary, genital, and alimentary tracts share a single conduit [5].

Cloaca malformation is characterized by the passage of meconium, stool, and urine through a single perineal opening. Prenatal diagnosis is intractable and tricky, and the ultrasound findings are vague and nonspecific; therefore, the MRI is preferred and highly recommended for diagnosis since it is more informative and accurate.

Bischoff et al. reported the frequency of abnormal prenatal ultrasound findings in patients with persistent cloaca as follows: abdominal cystic mass (41.1%), hydronephrosis (37.9%), oligohydramnios (24.2%), distended bowel (20%), ascites (15.8%), two-vessel cord (14.7%), dilated bladder (14.7%), dilated ureter (14.7%), polyhydramnios (10.5%), echogenic bowel (8.4%), multicystic kidneys (8.4%), hydrops fetalis (7.4%), hydrocolpos (4.2%), absent kidney (3.2%), abnormal spine (3.2%), and anorectal atresia (3.2%) [6].

When cloaca malformation is not treated properly and promptly, it can be lethal and dismal. It could be isolated or may be associated with other systems’ malformations and syndromes. Because the complications are severe and lethal, immediate and careful management is essential and lifesaving.

Because a fetus can die in the uterus from high-grade urinary obstruction [7], and a newborn can die from pulmonary hypoplasia and renal impairment induced by oligohydramnios [2,8-10], mental health support is needed for the mother, along with managing the fetus as a high-risk pregnancy [11].

Surgical correction of anatomical defects is always challenging as it is highly technical to reattain excellent functional anatomical repair, with the main target of ensuring fair bowel and urine control, fair sexual function, and fertility and renal function.

Persistent cloaca is a potentially devastating disease, necessitating multiple rounds of corrective surgery. Even with the most advanced treatments, there can be significant urological and gynecological sequelae, including incontinence and infertility [12]. Advanced, sophisticated, and varied surgical management could include single-session reconstructive surgery in the neonatal period or surgery scheduled later after initial drainage procedures in the first 48 hours of life.

To avoid severe and life-threatening complications of obstruction, colostomy should be considered first; however, the reconstructive repair surgery is usually postponed until the child is between the ages of six and 18 months.

Following the recommendation of the posterior sagittal approach, which promotes a clear vision of the anatomical defect, safe dissection, mobilization, and thus less muscle complex injury than in the abdominal approach, and less fecal, urine incontinence, which are the major complications encountered and been for a long time, a dilemma of repaired congenital malformations. The prevalence of fecal and urinary incontinence following posterior sagittal anorectoplasty for anorectal malformation has been widely observed and assessed, with many variations; according to a study by Ghorbanpoor et al., good fecal continence was observed in 91.3% of patients with low type anorectal malformation compared to 72.8% of patients with high type anorectal malformation; however, the difference was not significant [13]. Furthermore, researchers discovered that incontinence was less common in low abnormalities and more common in high or intermediate anomalies, whereas constipation was more common in low anomalies and less common in high and intermediate anomalies, according to Harjai et al. Thirty-one percent of the patients born with anorectal abnormalities who had posterior sagittal anorectoplasty (PSARP) were completely continent, 38% experienced fecal soiling, and 31% had constipation difficulties [14].

## Conclusions

Reconstructive surgery for the cloaca and anorectal malformations was significantly changed by PSARVUP or TUM approach, which is preferable, better, and safer than the abdominal approach for the cloaca and anorectal malformations. As the prognosis on bowl, urinary control, sexual function is better with the Pena technique. This approach facilitates a clear vision and is more obvious in the dissection, resulting in reduced neurovascular injury, the main issue of anorectal malformations. Fecal and urinary incontinence significantly decreased and guaranteed normal renal function and fertility. An experienced and skilled multidisciplinary team is essential; collaboration with specialists and mental health support is also highly recommended.
